# Two Sides to Every Story: Herpes Simplex Type-1 Viral Glycoproteins gB, gD, gH/gL, gK, and Cellular Receptors Function as Key Players in Membrane Fusion

**DOI:** 10.3390/v13091849

**Published:** 2021-09-16

**Authors:** Nithya Jambunathan, Carolyn M. Clark, Farhana Musarrat, Vladimir N. Chouljenko, Jared Rudd, Konstantin G. Kousoulas

**Affiliations:** School of Veterinary Medicine, Louisiana State University, Baton Rouge, LA 70803, USA; njambu1@lsu.edu (N.J.); cclar97@lsu.edu (C.M.C.); fmusar1@lsu.edu (F.M.); vchoul1@lsu.edu (V.N.C.); jrudd4@lsu.edu (J.R.)

**Keywords:** herpes simplex virus, membrane fusion, virus entry, glycoproteins, receptors, signaling, cell fusion

## Abstract

Herpes simplex virus type-1 (HSV-1) and type-2 (HSV-2) are prototypical alphaherpesviruses that are characterized by their unique properties to infect trigeminal and dorsal root ganglionic neurons, respectively, and establish life-long latent infections. These viruses initially infect mucosal epithelial tissues and subsequently spread to neurons. They are associated with a significant disease spectrum, including orofacial and ocular infections for HSV-1 and genital and neonatal infections for HSV-2. Viral glycoproteins within the virion envelope bind to specific cellular receptors to mediate virus entry into cells. This is achieved by the fusion of the viral envelope with the plasma membrane. Similarly, viral glycoproteins expressed on cell surfaces mediate cell-to-cell fusion and facilitate virus spread. An interactive complex of viral glycoproteins gB, gD/gH/gL, and gK and other proteins mediate these membrane fusion phenomena with glycoprotein B (gB), the principal membrane fusogen. The requirement for the virion to enter neuronal axons suggests that the heterodimeric protein complex of gK and membrane protein UL20, found only in alphaherpesviruses, constitute a critical determinant for neuronal entry. This hypothesis was substantiated by the observation that a small deletion in the amino terminus of gK prevents entry into neuronal axons while allowing entry into other cells via endocytosis. Cellular receptors and receptor-mediated signaling synergize with the viral membrane fusion machinery to facilitate virus entry and intercellular spread. Unraveling the underlying interactions among viral glycoproteins, envelope proteins, and cellular receptors will provide new innovative approaches for antiviral therapy against herpesviruses and other neurotropic viruses.

## 1. Introduction

Herpesviruses cause significant morbidity and mortality in humans and animals. There are more than 120 species of herpesvirus identified to date, including nine human herpesviruses [1]. The family *Herpesviridae* include herpesviruses of mammals, birds, and reptiles. They are divided into three subfamilies, alpha, beta, or gamma, based on their genetic sequences and their biological properties [1,2]. Herpes simplex viruses are the prototypic alphaherpesviruses that differ from the beta and gamma subfamilies by their ability to establish latency (a transcriptionally repressed state)—in the central and/or peripheral nervous systems of the host [3,4]. Latent viruses can reactivate from latency and spread to innervation sites, causing clinical symptoms. The hallmark of alphaherpesviruses is their ability to infect the nervous system and establish latency. The underlying mechanisms for neuronal infectivity remain poorly understood [3,5,6].

There are two genetically and serologically distinct human herpesviruses, HSV-1 and HSV-2. HSV-1 causes primarily orofacial and genital infections and rarely encephalitis [7,8,9]. The name herpes is from the ancient Greek word (ερπειν), which means “to creep” or “crawl” attributed to the tingling sensation in initial infections of mucosal/epithelial tissues before the formation of herpetic lesions. HSV-1 ocular infections are a major cause of infectious blindness in the US, and severe cases can necessitate corneal transplants. In the US, more than 400,000 people suffer from recurrent herpetic keratitis. HSV-2 causes predominantly genital infections and neonatal infections that can cause severe disease, fetus defects, and even death. There are an estimated 1500–2000 cases of neonatal infection each year. HSV-1 infects more than 3.7 billion people worldwide [10,11,12], and HSV-2 is estimated to infect 0.5 billion people globally. In the United States, these numbers are predicted to increase by >600,000 new infections per year by 2050 [10]. Initial infection of mucosal epithelium with either HSV-1 or HSV-2 results in viral replication and shedding followed by immunopathogenesis resulting in cold sores, blisters, and genital lesions [13].

Antiviral drugs including acyclovir, famciclovir, vidarabine, penciclovir, and valacyclovir are often used to treat HSV infections. While these medications reduce the severity and frequency of the symptoms, they do not cure the infection. Additionally, there is growing resistance to these drugs, particularly in immunocompromised patients, and many of these drugs have serious side effects [14]. Despite the current need, there is no available prophylactic or therapeutic vaccine for HSV-1 or HSV-2. This review focuses on the role of viral glycoproteins in membrane fusion events and the facilitation of virus entry and cell spread in mucosal/epithelial tissues and ganglionic neurons.

### 1.1. HSV-1 Lifecycle

HSV-1 initial infection occurs in epithelial cells before spreading to the trigeminal ganglia and establishing lifelong latency [15]. From latency, HSV-1 can reactivate and undergo lytic replication. The newly made virions are transported through the neuronal axons to epithelial cells, where infection manifests as cold sores or corneal keratitis. Alternatively, reactivation from latency may result in virion transport to the central nervous system, where it can cause meningitis and encephalitis [16]. HSV-1 can enter host cells by fusion of the viral envelope with the plasma membrane or endocytosis depending on the cell type [17,18]. Importantly, virion entry into neuronal axons is exclusively through plasma membrane fusion (reviewed in [19]). Upon attachment of the virion particle to cell surfaces, viral glycoproteins interact with the cellular receptors, which juxtapose the viral envelope with the plasma membrane. This juxtaposition facilitates the fusion of the two membranes resulting in the formation of a pore through which viral capsids and tegument proteins are released into the cytoplasm of infected cells. These capsids are then transported along the microtubules using cellular dynein motors to reach the nucleus, and the viral DNA is released into the nucleus, where it undergoes replication. Viral capsids are assembled within cellular nuclei and egress through a complicated process that involves primary envelopment of virion capsids, de-envelopment, and final re-envelopment. In this review, we examined the virion composition and the key viral and cellular proteins that are involved in facilitating virus entry into cells, ahead of discussing the relevant mechanisms that are involved in virus entry.

### 1.2. Virion Structure

Like other members of the *Herpesviridae* family, HSV-1 consists of a linear double-stranded DNA genome contained within an icosahedral capsid. The virion capsid is covered by a layer of proteins, called the tegument, containing more than 20 viral proteins. The entire virion particle is enclosed in a lipid bilayer envelope containing at least 20 viral proteins, of which 13 are glycoproteins [20]. The viral glycoproteins play essential roles in virion attachment to various cellular receptors, virus entry via either fusion of the viral envelope with the plasma membrane, or receptor-mediated endocytosis [17,21].

Although herpesvirus genomes can contain more than 100 genes, approximately 40 core genes are shared among all subfamilies of herpesvirus [22]. These genes code for structural components of the virion particles and proteins that serve essential functions in various aspects of the virus lifecycle, including viral replication, transcriptional regulation, and assembly and egress of enveloped virions out of infected cells. Most likely, these genes are responsible for the distinct biological properties of each subfamily that distinguish it from other subfamilies [3]. In this regard, glycoproteins and proteins embedded in the viral envelope are critical determinants of infectivity since they mediate virion attachment and entry into cells [17].

## 2. HSV-1 Viral Glycoproteins

### 2.1. Glycoprotein B (gB)

**Figure 1:**
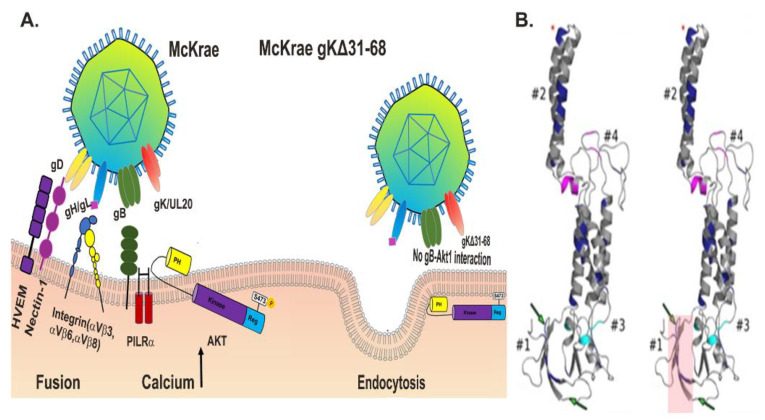
(**A**) Models of HSV-1 entry into host cells. The schematic depicts the interaction of viral glycoproteins gD, gH/gL, and gB with their cognate cellular receptors (HVEM (gD), nectin-1(gD), Integrins (gH/gL), PILRa (gB) and AKT (gB)). Membrane fusion is mediated by gB and regulated by complex protein interactions among gD, gH/gL, gK/UL20, and cellular receptors (left side). Deletion of the amino-terminal 31–68 aa of gK in the HSV-1 (McKrae), gKΔ31–68 virus prevents translocation of AKT to extracellular spaces and interaction with gB. This results in inhibition of virus entry via membrane fusion while allowing entry through receptor-mediated endocytosis (right side) [27]. (**B**) The predicted secondary structures of wild-type gK and gKΔ31–68 are shown (highlighted region depicts the actual deletion) oriented with the amino-terminal portion of gK proximal to cellular membranes. #1, #2, #3 and #4 corresponds to different domains of gk. The green arrows point to the predicted *N*-glycosylation sites. The conserved alpha-helical membrane-spanning domains, as well as the alphahelical domains conserved in the carboxyl-terminus of gK, are shown. Magenta and Cyan colors correspond to the alignment of gKs specified by HSV-1, HSV-2, Varicellar Zoster virus (VZV), and pseudorabies virus (PRV), as detailed previously [28]. Asterisk indicates YTK sequence in domain 2.

HSV-1(F) glycoprotein gB is one of the most conserved glycoproteins across all subfamilies of herpesviruses [23]. It is 904aa in length and functions as a class III membrane fusion protein, which combines characteristic features of class I and II fusion proteins similar to the vesicular stomatitis virus (VSV) fusion glycoprotein G [24,25,26]. Membrane fusion triggered by gB is highly regulated requiring the presence of the viral glycoproteins, gD, gH, and gL, which form the minimum “fusion machine” complex (Figure 1). Additional membranes glycoproteins, viral envelope proteins, and tegument proteins are involved in gB-mediated membrane fusion phenomena, most notably, glycoprotein K (gK) that binds and modifies gB-mediated membrane fusion (discussed in more detail later).

Glycoprotein gB has a spike-like ectodomain, a hydrophobic membrane domain, a transmembrane domain (TMD), and a cytoplasmic tail domain (CTD) [29]. Glycoprotein gB is essential for attachment and entry of the virion into different types of cells [30]. During entry, gB undergoes a series of conformational changes from its pre-fusion to its post-fusion state. These conformational changes expose hydrophobic peptides embed into the apposing membranes to initiate membrane fusion. The pre-fusion conformation of gB was recently elucidated, while the post-fusion conformation was resolved almost 10 years ago [31]. The fully folded form of the gB cytodomain forms a clamp-like structure that stabilizes the prefusion complex and prevents it from prematurely folding to a post-fusion form. Mutations in the cytoplasmic tail domain (CTD) of gB cause the clamp to destabilize, resulting in mutant viruses that are hyper-fusogenic forming multinucleated cells (syncytia) [32]. The conformational change in gB from pre-fusion to post-fusion is regulated by glycoprotein H (gH). The cytoplasmic tail of gH functions to destabilize the cytoplasmic domain clamp of gB, which causes a conformational change in gB that allows fusion to occur. Shorter gH cytoplasmic domains are known to reduce gB-mediated membrane fusion, most likely because they cannot function as efficient wedges to destabilize the clamp [33].

The alpha-helical domain within the carboxy terminus domain of gB regulates cell fusion, and deletions or mutations in the gB cytoplasmic terminus cause extensive fusion in both virus-induced and virus-free transient expression systems [34]. The gB ectodomain interacts with the ectodomain of gH and activates membrane fusion [34]. The membrane-proximal region (MPR) of gB is hydrophobic and lies between the ectodomain and the transmembrane domain (TMD) [17]. Certain amino acids in this MPR regulate gB’s fusogenicity by either facilitating or sequestering the fusion loops during the fusion reaction. Both the TMD and MPR are essential in enabling the lipid mixing and formation of the fusion pore once the fusion reaction has been initiated. Amino acids in the TMD are highly conserved among alphaherpesviruses [34,35].

### 2.2. Glycoprotein D (gD)

Glycoprotein gD is 369aa in length and functions as a type I membrane protein. gD has a single transmembrane domain and an N-terminal ectodomain of 316 residues with three N-linked glycosylation sites [36]. The C-terminal portion of the gD ectodomain is important for HSV-1 infectivity since it affects the binding of gD to cellular receptors [37]. Glycoprotein gD binds three classes of receptors in the host cell: (i) HVEM, which is a member of the tumor necrosis factor receptor superfamily (TNFR); (ii) HveC (nectin-1), a member of immunoglobulin (Ig) superfamily; and (iii) 3-O-sulphonated derivatives of heparan sulfate [38]. Most notably, gD is essential for binding nectin-1 and an important target for neutralizing antibodies [39].

### 2.3. Glycoprotein H (gH) and Glycoprotein L (gL)

Glycoprotein gH is an 838aa glycoprotein encoded by the UL22 gene. Like other type I glycoproteins, gH includes multiple domains: a signal peptide, an ectodomain, transmembrane domain, and cytoplasmic domain. Glycoprotein gL, a 224aa glycoprotein encoded by the UL1 gene, has a signal peptide but lacks a transmembrane domain and cannot associate with membranes independently. While UL1 (gL) and UL22 (gH) are regulated by different promotors, gH and gL are always found in a heterodimeric protein complex [40].

Glycoproteins gH and gL are highly conserved among all members of the *Herpesviridae* and serves critical roles in virus entry [40,41]. Deleting either or both gH and gL abrogates the heterodimer formation and results in a lethal phenotype where virions bind to the plasma membrane but cannot enter cells or induce virus-mediated cell-to-cell fusion [42,43]. The gH/gL heterodimer requires the expression of both proteins to fold and traffic properly through the cell. In the absence of gL, gH is structurally immature and retained in the endoplasmic reticulum [44]. Glycoprotein gL is, therefore, necessary as a chaperone to ensure proper folding of gH. In the absence of gH, gL cannot anchor in the membrane and is secreted by the cell. gL associates with the N terminal of gH, and they require each other for proper folding. gH is not able to reach the cell surface without gL. Transient expression results in both proteins being found together in the cell plasma membrane suggesting that no other viral proteins are required for the formation and trafficking of the gH/gL heterodimer.

While the gH/gL heterodimer is essential for viral entry, the virion can still attach to the cell membrane without the heterodimer, suggesting that these proteins are not involved in attachment or receptor binding. X-ray crystallographic structure analysis has failed to identify any structural feature to indicate that gH/gL serves a direct role in fusion [41]. The amino termini of gH and gL are less conserved and might have evolved to support species-specific glycoprotein interactions [44]. The ectodomain of gD interacts with the homotypic functional interaction site of gH/gL [45]. Moreover, gD forms a protein complex with gB in the absence of gH/gL, as well as with gH/gL in the absence of gB [46] (discussed later under mechanisms of virus entry). Although gH and gL are highly conserved among different herpes viruses, only gH/gL of HSV-1 and HSV-2 function interchangeably, suggesting that their functions in membrane fusion phenomena are virus and species-specific [47,48]. In contrast, Saimiriine herpesvirus 1 (SaHV-1) is an alphaherpesvirus family related to HSV-1 and HSV-2 [49]. Glycoprotein gB homologs from HSV-1 and SaHV-1 were interchangeable, although neither gD nor gH/gL were interchangeable, suggesting that their functional association is type-specific [50].

### 2.4. Glycoprotein K(gK)

Glycoprotein K is essential for viral assembly and egress. Specifically, gK regulates membrane fusion during virus entry and virus-induced cell fusion. gK physically binds gB and gH and modulates gB-mediated membrane fusion. The gK/UL20 complex physically binds to the amino terminus of gB and regulates its fusogenecity by binding at both the intracellular and extracellular domains [51,52]. The N-glycosylation sites at the amino terminus of gK are highly conserved and contribute to the overall structure of the gK amino terminus and its interaction with the amino terminus of gB. The many syncytial mutations in gK suggest a role for gK as a negative regulator of fusion. Mutation at the N glycosylation site N58A of the amino terminus causes extensive fusion [53]. However, deletion of the amino-terminal gK amino acids 31–68 abrogated virus-induced cell fusion caused by the gBΔ28syn carboxyl-terminal deletion in gB (deletion of the carboxyl-terminal 28 aa that causes extensive syncytia formation), indicating that the amino terminus of gK is required for gB-mediated cell fusion [51]. Thus, gK has both positive and negative regulatory functions in gB-mediated membrane fusion phenomena. The HSV-1 live-attenuated vaccine strain VC2 includes deletions of both gK glycosylation sites (ΔgK 31–68) as well as ΔUL20 4–22. In VC2, gK and UL20 are therefore unable to bind the fusion complex, and the virion cannot enter via fusion. VC2 produces robust immune responses compared to its parental strain HSV-1(F) [54,55,56,57]. The amino terminus of gK functions as a critical determinant for virus infectivity into neuronal axons. Specifically, deletion of the amino terminus of HSV-1 (McKrae) gK (31–68) prevents the virion from entering via fusion but does not impact entry via endocytosis [58,59]. Although gK may not directly bind to HSV-1 receptors, overexpression of gK increases expression of 3-OS-HS, PILR alpha, nectin-1, and nectin-2 receptors and severity of corneal scarring, highlighting its role in viral pathogenesis [59]. gK is highly conserved among all neurotropic alphaherpesviruses, and thus, it is hypothesized to play important roles in neuronal infection and neuropathogenesis [28]. HSV mutant viruses overexpressing gK were reported to exacerbate mouse corneal scaring attributed at least in part to an amino-terminal 8 amino acid peptide. Importantly, gK-induced corneal scarification was dependent on the binding of gK to the signal peptide peptidase (SPP) [60,61,62,63]. In addition, UL20 is found in a heterodimeric form with gK GODZ (DHHC3), and this interaction is required for gK-induced pathology [64].

## 3. HSV Receptors and Cell Tropism

HSV-1 has a wide host range able to productively infect a variety of cell types based on the combination of cellular receptors available for both gD and gB. The availability of cellular receptors is critically important to understand the host and tissue range of the virus. HSV-1 can infect certain regions of the brain, primarily the hippocampus in the temporal lobe [65]. In human adult brains, three receptors are differentially expressed in the hippocampus: MYH9 (gB receptor), nectin-1 (gD receptor), and HVEM (gD receptors). PILRα, another gB receptor, is most amply expressed in the human cerebellum [66]. This concurrent expression of gD and gB receptors may contribute to the tropism of HSV-1 in postnatal brain tissues. *In utero*, HSV-1 exposure to the developing fetus does not produce any adverse pathology likely due to the low levels of receptor expression in the fetal brain. Alternatively, infants may exhibit severe disease postnatally, including encephalopathy [66]. These differences in receptor expression between developmental stages highlight the relevance to cellular receptors and tissue tropism to understand HSV-1 disease processes. The glycan moieties of viral glycoproteins also play a role in many aspects of the viral life cycle. Glycans are involved in viral binding, entry, transmission, and evasion of the host immune system. Thus, it is likely that glycosylation of gB plays an essential role in viral pathogenesis [67].

Glycoprotein gB forms a stable trimer in its pre-fusion state and undergoes a drastic conformational change by interactions with gD/gH/gL and cellular receptors [68,69]. gB-mediated membrane fusion is also facilitated by direct interactions of gB with certain cellular receptors, including the non-muscle myosin heavy chain (NMHC-IIA) and the inhibitory immunoreceptor paired immunoglobin-like type 2 receptor α (PILR-α) [35,70,71]. The ability of gB to bind with the PILR α receptor is an essential factor in viral pathogenicity. The highly pathogenic strain HSV-1 (McKrae) entered Chinese hamster ovary (CHO) cells expressing human PILRα more efficiently than the lab-adapted wild-type strain HSV-1 (F). This entry difference was attributed to sequence differences in the amino termini of gB. The amino terminus of gB binds to the PILR α receptor and results in enhanced virion entry via gB-mediated fusion of the viral envelope [72]. Thus, the tropism is regulated at multiple levels by (i) co-expression of receptors for gD and gB, (ii) the class of receptors, and the (iii) the amino terminus of gB.

### 3.1. Nectin-1

Nectin-1 is a cellular adhesion molecule ubiquitously found in a wide range of tissues, including epithelial tissues and the chemical synapse of neuronal tissue [73]. It functions in the formation and organization of adherens and tight junctions in a Ca 2+ independent manner. Nectin-1 and herpesvirus entry mediator (HVEM) are entry receptors for both HSV-1 and HSV-2. Nectins 1 and 2 have 30% similarity, while nectin-1 is a better receptor for HSV-2 [74]. However, some HSV-1 strains isolated from cases of encephalitis utilize the nectin-2 receptor. Double or triple mutations at aa 222, 223, and 215 (R222N/F223I/D215G) of gD, abrogated fusion/entry activity using nectin-1 and nectin-2 receptors, and a mutant virus with these mutations was not able to infect human epithelial and neuronal cell lines [75]. However, mutations that abrogate the utilization of other receptors by gD for fusion and entry activity maintain the ability to infect cells through nectin-1 and nectin-2.

Nectin-1 is expressed in a variety of human tissues such as ganglia, trachea, prostate, thyroid, and the central nervous system. Cell lines such as keratinocytes, human corneal epithelium, retinal pigment epithelium, human conjunctival epithelium, fibroblasts, and neuroblastomas express nectin-1. Nectin-1 expression is a biomarker for metastatic breast cancer and highly migratory and invasive squamous cell carcinoma. It is upregulated in pediatric embryonal tumors and brain tumors. Therefore, these cell types can be easily targeted by oncolytic herpes viruses (oHSV). Pediatric brain tumors are ideal targets for oHSV, while nectin-1 expression is a valuable biomarker to predict the patient’s response to oHSV [76].

### 3.2. Herpes Virus Entry Mediator (HVEM)

HVEM is also known as tumor necrosis factor receptor superfamily-14 (TNFRSF14), tumor necrosis factor receptor-like 2 (TR2), and CD antigen CD270. It is a type I integral membrane protein, which is involved with immune-regulatory signal transduction pathway proteins [77]. The HVEM cytoplasmic domain binds to TNFR- associated factor family members leading to the activation of NF-κB, Jun N-terminal kinase, and AP-1 leading to T cell activation, proliferation, cytokine release, and expression of cell surface activation markers [78,79]. HVEM is expressed on T and B lymphocytes and other leukocytes, epithelial cells, fibroblasts, and tissues of the lung, liver, kidney, and to a lesser extent, the brain tissue [77]. LIGHT, lymphotoxin—α (LTα), the immunoglobulin domain-containing cell-surface receptors B and T lymphocyte attenuator (BTLA), and CD160 are natural HVEM ligands [80]. HSV-1 entry into activated T cells is thought to be mediated by HVEM, although HVEM does not appear to be a major receptor for virus entry in most other cells [77]. Specific gD mutations encoded by the rid1and ANG virus strains reduce infectivity on HVEM-expressing cells [77]. The interacting region of gD with HVEM involves amino acids 7 to 15 and 24 to 32 within the N-terminal hairpin loop [81].

### 3.3. PILR-α

PILR-α is found in myeloid cells and neural tissues. Recombinant viruses with the gB-T53A and gB-T53/480A mutations exhibited phenotypes similar to wild-type viruses, except that they were defective in PILR-α-dependent viral entry. These mutant viruses exhibited a significant reduction in viral replication in the eyes, stromal keratitis, and neuroinvasiveness in mice [67,82]. PILR-α-dependent viral entry requires gB O-glycosylation, which may play a significant role in viral replication, pathogenesis, and neuroinvasiveness in vivo [83]. PILR-α is expressed in macrophages and dendritic cells of the immune system [84,85]. Therefore, infection of these cells by HSV-1 can modulate their activity leading to attenuation of innate and adaptive immune responses against HSV-1.

### 3.4. The αvβ6 and αvβ8 Integrin Receptors

The αvβ8 integrin receptors interact with the gH/gL heterodimer (Figure 1). This interaction enables both virus entry and endocytosis into acidic endosomes [86]. αvβ6- and αvβ8 integrins promote dissociation of gL from the gH/gL complex, and this causes activation of the fusion complex [87]. The binding of αvβ3 integrins by gH may facilitate virus entry into specific cell types in vivo. Several other viruses use integrins as cellular receptors, including adenovirus, rotavirus, parechovirus 1, and hantavirus [88]. Moreover, human herpesvirus 8 (HHV8) binds α3β1 integrins via an RGD sequence present in gB. αvβ3 integrins interact with gH through its RGD (Arg-Gly-Asp) integrin-binding motif [89]. HSV and human cytomegalovirus (hCMV) activate AKT during virus entry [90]. Relocalization of AKT to the outer leaflet of the plasma membrane increases the availability of αvβ3 integrins for gH binding facilitating membrane fusion. gH–αvβ3 binding is required for the substantial increase in intracellular calcium concentrations by triggering the release of endoplasmic reticulum calcium stores [91,92]. The αvβ8 integrin receptors, while not essential, clearly play a role in intracellular calcium signaling during entry.

### 3.5. 3-O-Sulfated Heparan Sulfate (3-OS HS)

Heparan sulfate (HS) is present on all cell surfaces in a variety of polysaccharide structures that physically alter their localization and functional properties [93]. These polysaccharides serve as initial receptors for many viruses, including all human and animal herpesviruses, except the Epstein–Barr virus [94]. HSV-1 utilizes HS to facilitate membrane fusion by binding to gD and gB and may serve important functions in facilitating entry into corneal fibroblasts [89,94,95,96].

## 4. Mechanistic Aspects of HSV-1 Entry

HSV-1-mediated membrane fusion of the viral envelope with plasma membranes during virus entry and virus-induced cell fusion facilitates virus spread. They are mediated by viral glycoproteins within the viral envelope and on infected cell surfaces [21]. Viral entry may occur at neutral pH conditions in which the viral envelope fuses with plasma membranes allowing virion capsids to enter the cytoplasm of infected cells. This pH-independent entry is utilized in many different cell types of epithelial or fibroblastic origin [18,97,98,99,100]; notably, it is used exclusively for virus entry into neuronal axons [18,101,102]. An alternative mechanism for HSV-1 entry into cells involves receptor-mediated endocytosis placing enveloped virions within endosomes. The low-pH environment of endosomes causes fusion of the viral envelope with endosomal membranes resulting in the cytoplasmic deposition of virion capsids [18,103]. Depending on the cell type, HSV-1 may preferentially utilize one of these entry mechanisms or both at the same time [30,98,104,105,106,107,108,109]. The virus spreads from infected to uninfected cells by virus-induced cell fusion, enabling virions to spread into uninfected cells, avoiding extracellular spaces.

### 4.1. Interactive Protein Complexes of Viral Glycoproteins and Cellular Receptors Function in Virus Entry

HSV-1 attaches to the plasma membrane by glycoproteins B (gB) and C (gC) via transient interactions with glycosaminoglycans (GAG) of cell surface proteoglycans, especially heparan sulfate [94,110,111]. Glycoprotein gB interacts with the paired immunoglobulin-like type 2 receptor alpha (PILR), the non-muscle myosin heavy chain IIA (NMHC-IIA), and the myelin-associated glycoprotein (MAG) to mediate virus entry. Glycoprotein gD facilitates virus entry by binding to the herpesvirus entry mediator (HVEM, also called HveA), nectin-1 (HveC), and 3-O-sulfated heparan sulfate [70,71,95,112,113,114,115]. HSV glycoproteins gD, gB, and the heterodimer gH/gL constitute the minimum membrane fusion protein complex that functions in virus entry and virus-induced cell fusion. Following the binding of gD and gB, a conformational change in gB is transduced through altered interaction of gH/gL with gB, triggering membrane fusion [42,116,117,118,119,120]. gB forms a stable trimer in its pre-fusion state and undergoes a drastic conformational change in response to interactions with gD/gH/gL and cellular receptors [68,69].

For both HSV-1 and HSV-2, the initial binding of gD to cellular receptors causes conformational changes in gB through the gH/L complex (reviewed in [102]). This cascade of sequential interactions among gD, gH/gL, and gB is specific to HSV since it is not required in other alphaherpesviruses. Specifically, for pseudorabies virus (PRV), PRV gD is not required to interact with cellular receptors, while in varicella-zoster virus (VZV), there is no gD ortholog [121]. The gH/gL heterodimer modulates gB’s fusogenicity in a cascade manner by transducing signals received from interactions of gD with a-V integrins [32,50,87].

### 4.2. The Intriguing Role of gK in Virus Entry

The virion particle can be considered as an interactive protein complex since most glycoproteins embedded within the viral envelope interact with each other as well as with several tegument proteins. Thus, it is not surprising that certain viral glycoproteins, membrane proteins, and tegument proteins may alter virus entry. Specifically, glycoprotein M (gM) prevents the fusion of nascent virions released from infected cells and is required for membrane fusion through interactions with gK [122,123]. gK is an intriguing modulator of membrane fusion phenomena since its amino terminus is essential for virus entry by fusion of the viral envelope with the plasma membrane and neuronal axons, indicating a highly conserved function that is specific to neurotropic viruses (Figure 1) [27,28,124,125]. gK may modulate gB-mediated membrane fusion through interactions with gB and the gD/gH/gL/gM protein complex and interactions with cellular receptors.

### 4.3. Virus-Induced Cell Fusion

The core fusion machinery of herpesviruses composed of gB, gD, and gH/gL is required for virus entry and virus-induced cell-cell fusion [35,40,81,126,127,128,129,130,131]. However, virus-induced cell fusion requires the expression of viral glycoproteins on cell surfaces and the presence of additional viral glycoproteins, including gE, gI, gM, gK, and the UL20 and UL45 proteins [51,132,133,134,135,136]. In addition, the tegument proteins UL11, UL16, and UL21 bind to the cytoplasmic tail of gE modulating cell fusion [137]. This differential requirement of viral proteins and glycoproteins in virus entry versus virus-induced cell fusion may stem from the different energy requirements for membrane fusion of the viral envelope with the plasma membrane versus fusion among cells.

### 4.4. Role of HSV-1 Glycoprotein K (gK) in Membrane Fusion

The UL20 and UL53 (gK) genes are among the most conserved alphaherpesvirus genes encoding 222 and 338 amino acids, respectively, each with four membrane-spanning domains having opposite orientations within membranes with gK having both amino and carboxyl termini extracellularly, while UL20 has both amino and carboxyl termini intracellularly [138,139,140,141,142]. HSV-1 gK and UL20 physically interact and function to coordinate intracellular transport, cell-surface expression, and mediate functions in virus-induced cell fusion, virus entry, virion envelopment, and egress from infected cells [125,135,136,141,143,144,145,146,147,148,149]. Both gK and UL20 are components of the virion envelope [125,150,151]. The gK/UL20 protein complex interacts with gB and gH and is required for gB-mediated cell fusion [51,52]. HSV-1 gK is a structural component of virions and functions in virion entry [124,148]. The gK/UL20 protein complex binds to gB and gH and is required for gB-mediated cell fusion [51,52]. A 39 amino-terminal deletion of gK inhibits virus-induced cell-to-cell fusion and virus entry into neuronal axons, but not other cell types (fibroblasts, epithelial cells) without drastically inhibiting virion envelopment and egress [58,152,153]. The human ocular clinical strain HSV-1(McKrae) gK∆31–68 with a deletion of 37 aa failed to infect mouse trigeminal ganglia after ocular infection of scarified mouse eyes [58], indicating that the amino terminus of gK plays a pivotal role in corneal infection and neuroinvasiveness [58,153]. Mutations of gK result in viruses with domain-specific defects in entry, assembly, or egress, indicating gK domains that function in membrane fusion and virion assembly are functionally distinct and genetically separable [27,147,149,154,155,156]. Molecular evolution modeling of the 3-dimensional predicted structure of gK has confirmed that conserved domains across alphaherpesviruses have lower evolution rates, while those that may be involved in cellular tropisms, such as extracellular domains, have higher evolution rates [28].

### 4.5. Cell Signaling in Membrane Fusion

Akt is a serine/threonine kinase that functions in multiple cellular signaling pathways [157]. The three isoforms of Akt: Akt1, Akt2, and Akt3 share an N-terminal pleckstrin homology domain, a kinase domain, and a C-terminal regulatory domain [158]. Akt-1 is expressed in all tissues, including neurons, while Akt-2 and Akt-3 are expressed mostly in insulin-responsive tissues and brain or testes, respectively [159]. Recruitment of Akt-1 to plasma membranes and phosphorylation at S473 and T308 amino acids is initiated after signaling through cellular receptors activates the phosphoinositide-3-kinase (PI3K) signaling pathway [160,161,162,163,164,165]. Several viral proteins regulate cell survival, growth, apoptosis, inflammation, cell motility, and calcium signaling by modulating the PI3K/Akt signaling pathway [166]. These regulatory mechanisms play significant roles in multiple steps of the HSV-1 lifecycle, including viral entry, replication, latency, and reactivation from latency [90,167].

Glycoprotein gB binds to phosphorylated Akt during virus entry [91], and HSV-1 entry induces intracellular calcium release [167]. Evidence for the equine herpesvirus-1 (EHV-1) showed that binding of gH to cellular integrin (α4β1) triggers intracellular calcium signaling and expression of phosphatidylserine on extracellular plasma membranes to facilitate EHV-1 entry suggesting that gH/gL may also be involved in calcium mobilization and plasma membrane flipping [168,169]. gK is required to bind gB to AKT, leading to its phosphorylation and thus triggering calcium release. Deleting gK31–68 forces the virion to enter exclusively by endocytosis, while the wild-type virion enters into neuronal axons exclusively by fusion (Figure 1). This result suggests that the amino terminus of gK and its binding partner UL20 interact and regulate the fusion protein gB during viral entry. The silencing of AKT with siRNA or an HSV-2 variant with a deletion in gB or gD either prevented calcium responses or phosphorylation of AKT, which inhibited virus entry [91].

The importance of intracellular calcium mobilization was also demonstrated by the fact that the Akt inhibitor miltefosine inhibited calcium signaling and virus entry into cells [91]. Importantly, deletion of the gK31–68 amino acids prevented HSV-1 from entering neuronal axons, most likely because it prevented interactions of gB with Akt. However, the gK31–68 mutant virus also did not mobilize intracellular calcium and failed to expose phosphatidylserine to extracellular membranes, suggesting that gK may exert regulatory effects on both gB and other viral glycoproteins, as well as on intracellular signaling through interactions with unknown cellular receptors [27,170].

## 5. Conclusions

Herpes simplex viruses have evolved a membrane fusion machinery exquisitely regulated for optimum infectivity and spread. This fusion machinery evolved to deal with infection of mucosal/epithelial tissues and, even more importantly, neurons. Viral proteins and glycoproteins conserved among neurotropic alphaherpesviruses are very likely to play important roles in virus entry into neuronal axons and retrograde transport to neuronal cell bodies where the virus establishes latency. Thus far, gK is the only glycoprotein essential for virus entry by fusion of the viral envelope with the plasma membrane (Figure 1). The fact that the gK/UL20 heterodimer is conserved among neurotropic alphaherpesviruses strongly suggests that this protein complex serves as a critical determinant of neuronal infection. The absence of gK/UL20 in betaherpeviruses and gammaherpesviruses suggests that entry via fusion vs. endocytosis is regulated through a different mechanism. Many questions remain to be answered in the quest to better understand the membrane fusion mechanisms that govern HSV entry, especially into neuronal axons. Both viral and cellular proteins are likely involved in orchestrating a cascade of events, including intracellular signaling, that facilitates membrane fusion. Uncovering the underlying mechanisms may provide a greater understanding of the sophisticated mechanisms that the virus has acquired over many thousands of years to deal with infection of highly sensitive neuronal endings in a “stealth” manner, where the virus downregulates any cellular antiviral response. Elucidation of these mechanisms may provide new innovative approaches for antiviral therapy against herpesviruses and other neurotropic viruses.

## References

[1] Davison A.J. (2002). Evolution of the herpesviruses. Vet. Microbiol..

[2] Davison A.J., Eberle R., Ehlers B., Hayward G.S., McGeoch D.J., Minson A.C., Pellett P.E., Roizman B., Studdert M.J., Thiry E. (2009). The order Herpesvirales. Arch. Virol..

[3] Roizman B., Knipe D., Whitley R.J., David M., Knipe P.H. (2006). Herpes Simplex Viruses. Fields Virology.

[4] Stanfield B., Kousoulas K.G. (2015). Herpes Simplex Vaccines: Prospects of Live-attenuated HSV Vaccines to Combat Genital and Ocular infections. Curr. Clin. Microbiol. Rep..

[5] Singh N., Tscharke D.C. (2020). Herpes Simplex Virus Latency Is Noisier the Closer We Look. J. Virol..

[6] Cohen J.I. (2020). Herpesvirus latency. J. Clin. Investig..

[7] Durukan D., Fairley C.K., Bradshaw C.S., Read T.R.H., Druce J., Catton M., Caly L., Chow E.P.F. (2019). Increasing proportion of herpes simplex virus type 1 among women and men diagnosed with first-episode anogenital herpes: A retrospective observational study over 14 years in Melbourne, Australia. Sex. Transm. Infect..

[8] Magdaleno-Tapial J., Hernandez-Bel P., Valenzuela-Onate C., Ortiz-Salvador J.M., Garcia-Legaz-Martinez M., Martinez-Domenech A., Perez-Pastor G., Esteve-Martinez A., Zaragoza-Ninet V., Sanchez-Carazo J.L. (2020). Genital Infection With Herpes Simplex Virus Type 1 and Type 2 in Valencia, Spain: A Retrospective Observational Study. Actas Dermosifiliogr..

[9] Spicknall I.H., Flagg E.W., Torrone E.A. (2021). Estimates of the Prevalence and Incidence of Genital Herpes, United States, 2018. Sex. Transm. Dis..

[10] James C., Harfouche M., Welton N.J., Turner K.M., Abu-Raddad L.J., Gottlieb S.L., Looker K.J. (2020). Herpes simplex virus: Global infection prevalence and incidence estimates, 2016. Bull. World Health Organ..

[11] Looker K.J., Magaret A.S., May M.T., Turner K.M., Vickerman P., Gottlieb S.L., Newman L.M. (2015). Global and Regional Estimates of Prevalent and Incident Herpes Simplex Virus Type 1 Infections in 2012. PLoS ONE.

[12] Looker K.J., Magaret A.S., Turner K.M., Vickerman P., Gottlieb S.L., Newman L.M. (2015). Global estimates of prevalent and incident herpes simplex virus type 2 infections in 2012. PLoS ONE.

[13] Whitley R.J., Kimberlin D.W., Roizman B. (1998). Herpes simplex viruses. Clin. Infect. Dis..

[14] Rechenchoski D.Z., Faccin-Galhardi L.C., Linhares R.E.C., Nozawa C. (2017). Herpesvirus: An underestimated virus. Folia Microbiol..

[15] Sharthiya H., Seng C., Van Kuppevelt T.H., Tiwari V., Fornaro M. (2017). HSV-1 interaction to 3-O-sulfated heparan sulfate in mouse-derived DRG explant and profiles of inflammatory markers during virus infection. J. Neurovirol..

[16] Steiner I., Benninger F. (2013). Update on herpes virus infections of the nervous system. Curr. Neurol. Neurosci. Rep..

[17] Madavaraju K., Koganti R., Volety I., Yadavalli T., Shukla D. (2020). Herpes Simplex Virus Cell Entry Mechanisms: An Update. Front. Cell Infect. Microbiol..

[18] Nicola A.V. (2016). Herpesvirus Entry into Host Cells Mediated by Endosomal Low pH. Traffic.

[19] Miranda-Saksena M., Denes C.E., Diefenbach R.J., Cunningham A.L. (2018). Infection and Transport of Herpes Simplex Virus Type 1 in Neurons: Role of the Cytoskeleton. Viruses.

[20] Arduino P.G., Porter S.R. (2008). Herpes Simplex Virus Type 1 infection: Overview on relevant clinico-pathological features. J. Oral Pathol. Med..

[21] Roizman B., Knipe D.M., Knipe D.M., Howley P.M. (2001). Herpes Simplex Viruses and Their Replication. Fields Virology.

[22] King A.M.Q., Adams M.J., Carstens E.B., Lefkowitz E.J., Family—Alloherpesviridae (2012). Virus Taxonomy.

[23] Zmasek C.M., Knipe D.M., Pellett P.E., Scheuermann R.H. (2019). Classification of human Herpesviridae proteins using Domain-architecture Aware Inference of Orthologs (DAIO). Virology.

[24] Hannah B.P., Cairns T.M., Bender F.C., Whitbeck J.C., Lou H., Eisenberg R.J., Cohen G.H. (2009). Herpes simplex virus glycoprotein B associates with target membranes via its fusion loops. J. Virol..

[25] Vollmer B., Prazak V., Vasishtan D., Jefferys E.E., Hernandez-Duran A., Vallbracht M., Klupp B.G., Mettenleiter T.C., Backovic M., Rey F.A. (2020). The prefusion structure of herpes simplex virus glycoprotein B. Sci. Adv..

[26] Roche S., Albertini A.A., Lepault J., Bressanelli S., Gaudin Y. (2008). Structures of vesicular stomatitis virus glycoprotein: Membrane fusion revisited. Cell. Mol. Life Sci..

[27] Musarrat F., Jambunathan N., Rider P.J.F., Chouljenko V.N., Kousoulas K.G. (2018). The Amino Terminus of Herpes Simplex Virus 1 Glycoprotein K (gK) Is Required for gB Binding to Akt, Release of Intracellular Calcium, and Fusion of the Viral Envelope with Plasma Membranes. J. Virol..

[28] Rider P.J.F., Coghill L.M., Naderi M., Brown J.M., Brylinski M., Kousoulas K.G. (2019). Identification and Visualization of Functionally Important Domains and Residues in Herpes Simplex Virus Glycoprotein K(gK) Using a Combination of Phylogenetics and Protein Modeling. Sci. Rep..

[29] Pellett P.E., Kousoulas K.G., Pereira L., Roizman B. (1985). Anatomy of the herpes simplex virus 1 strain F glycoprotein B gene: Primary sequence and predicted protein structure of the wild type and of monoclonal antibody-resistant mutants. J. Virol..

[30] Karasneh G.A., Shukla D. (2011). Herpes simplex virus infects most cell types in vitro: Clues to its success. Virol. J..

[31] Connolly S.A., Jardetzky T.S., Longnecker R. (2021). The structural basis of herpesvirus entry. Nat. Rev. Microbiol..

[32] Silverman J.L., Greene N.G., King D.S., Heldwein E.E. (2012). Membrane requirement for folding of the herpes simplex virus 1 gB cytodomain suggests a unique mechanism of fusion regulation. J. Virol..

[33] Rogalin H.B., Heldwein E.E. (2015). Interplay between the Herpes Simplex Virus 1 gB Cytodomain and the gH Cytotail during Cell-Cell Fusion. J. Virol..

[34] Foster T.P., Melancon J.M., Kousoulas K.G. (2001). An alpha-helical domain within the carboxyl terminus of herpes simplex virus type 1 (HSV-1) glycoprotein B (gB) is associated with cell fusion and resistance to heparin inhibition of cell fusion. Virology.

[35] Atanasiu D., Whitbeck J.C., de Leon M.P., Lou H., Hannah B.P., Cohen G.H., Eisenberg R.J. (2010). Bimolecular complementation defines functional regions of Herpes simplex virus gB that are involved with gH/gL as a necessary step leading to cell fusion. J. Virol..

[36] Watson R.J., Weis J.H., Salstrom J.S., Enquist L.W. (1982). Herpes simplex virus type-1 glycoprotein D gene: Nucleotide sequence and expression in Escherichia coli. Science.

[37] Fan Q., Kopp S., Connolly S.A., Muller W.J., Longnecker R. (2017). Mapping sites of herpes simplex virus type 1 glycoprotein D that permit insertions and impact gD and gB receptors usage. Sci. Rep..

[38] Carfi A., Willis S.H., Whitbeck J.C., Krummenacher C., Cohen G.H., Eisenberg R.J., Wiley D.C. (2001). Herpes simplex virus glycoprotein D bound to the human receptor HveA. Mol. Cell.

[39] Para M.F., Parish M.L., Noble A.G., Spear P.G. (1985). Potent neutralizing activity associated with anti-glycoprotein D specificity among monoclonal antibodies selected for binding to herpes simplex virions. J. Virol..

[40] Heldwein E.E. (2016). gH/gL supercomplexes at early stages of herpesvirus entry. Curr. Opin. Virol..

[41] Stampfer S.D., Heldwein E.E. (2013). Stuck in the middle: Structural insights into the role of the gH/gL heterodimer in herpesvirus entry. Curr. Opin. Virol..

[42] Hutchinson L., Browne H., Wargent V., Davis-Poynter N., Primorac S., Goldsmith K., Minson A.C., Johnson D.C. (1992). A novel herpes simplex virus glycoprotein, gL, forms a complex with glycoprotein H (gH) and affects normal folding and surface expression of gH. J. Virol..

[43] Roop C., Hutchinson L., Johnson D.C. (1993). A mutant herpes simplex virus type 1 unable to express glycoprotein L cannot enter cells, and its particles lack glycoprotein H. J. Virol..

[44] Fan Q., Longnecker R., Connolly S.A. (2015). A Functional Interaction between Herpes Simplex Virus 1 Glycoprotein gH/gL Domains I and II and gD Is Defined by Using Alphaherpesvirus gH and gL Chimeras. J. Virol..

[45] Fan Q., Longnecker R. (2010). The Ig-like v-type domain of paired Ig-like type 2 receptor alpha is critical for herpes simplex virus type 1-mediated membrane fusion. J. Virol..

[46] Gianni T., Amasio M., Campadelli-Fiume G. (2009). Herpes simplex virus gD forms distinct complexes with fusion executors gB and gH/gL in part through the C-terminal profusion domain. J. Biol. Chem..

[47] Muggeridge M.I. (2000). Characterization of cell-cell fusion mediated by herpes simplex virus 2 glycoproteins gB, gD, gH and gL in transfected cells. J. Gen. Virol..

[48] Cairns T.M., Landsburg D.J., Whitbeck J.C., Eisenberg R.J., Cohen G.H. (2005). Contribution of cysteine residues to the structure and function of herpes simplex virus gH/gL. Virology.

[49] Tyler S., Severini A., Black D., Walker M., Eberle R. (2011). Structure and sequence of the saimiriine herpesvirus 1 genome. Virology.

[50] Fan Q., Longnecker R., Connolly S.A. (2014). Substitution of herpes simplex virus 1 entry glycoproteins with those of saimiriine herpesvirus 1 reveals a gD-gH/gL functional interaction and a region within the gD profusion domain that is critical for fusion. J. Virol..

[51] Chouljenko V.N., Iyer A.V., Chowdhury S., Chouljenko D.V., Kousoulas K.G. (2009). The amino terminus of herpes simplex virus type 1 glycoprotein K (gK) modulates gB-mediated virus-induced cell fusion and virion egress. J. Virol..

[52] Chouljenko V.N., Iyer A.V., Chowdhury S., Kim J., Kousoulas K.G. (2010). The herpes simplex virus type 1 UL20 protein and the amino terminus of glycoprotein K (gK) physically interact with gB. J. Virol..

[53] Rider P.J.F., Naderi M., Bergeron S., Chouljenko V.N., Brylinski M., Kousoulas K.G. (2017). Cysteines and N-Glycosylation Sites Conserved among All Alphaherpesviruses Regulate Membrane Fusion in Herpes Simplex Virus 1 Infection. J. Virol..

[54] Bernstein D.I., Pullum D.A., Cardin R.D., Bravo F.J., Dixon D.A., Kousoulas K.G. (2019). The HSV-1 live attenuated VC2 vaccine provides protection against HSV-2 genital infection in the guinea pig model of genital herpes. Vaccine.

[55] Naidu S.K., Nabi R., Cheemarla N.R., Stanfield B.A., Rider P.J., Jambunathan N., Chouljenko V.N., Carter R., Del Piero F., Langohr I. (2020). Intramuscular vaccination of mice with the human herpes simplex virus type-1(HSV-1) VC2 vaccine, but not its parental strain HSV-1(F) confers full protection against lethal ocular HSV-1 (McKrae) pathogenesis. PLoS ONE.

[56] Stanfield B.A., Pahar B., Chouljenko V.N., Veazey R., Kousoulas K.G. (2017). Vaccination of rhesus macaques with the live-attenuated HSV-1 vaccine VC2 stimulates the proliferation of mucosal T cells and germinal center responses resulting in sustained production of highly neutralizing antibodies. Vaccine.

[57] Stanfield B.A., Rider P.J.F., Caskey J., Del Piero F., Kousoulas K.G. (2018). Intramuscular vaccination of guinea pigs with the live-attenuated human herpes simplex vaccine VC2 stimulates a transcriptional profile of vaginal Th17 and regulatory Tr1 responses. Vaccine.

[58] Saied A.A., Chouljenko V.N., Subramanian R., Kousoulas K.G. (2014). A replication competent HSV-1(McKrae) with a mutation in the amino-terminus of glycoprotein K (gK) is unable to infect mouse trigeminal ganglia after cornea infection. Curr. Eye Res..

[59] Allen S.J., Mott K.R., Ghiasi H. (2014). Overexpression of herpes simplex virus glycoprotein K (gK) alters expression of HSV receptors in ocularly-infected mice. Investig. Ophthalmol. Vis. Sci..

[60] Jaggi U., Wang S., Tormanen K., Matundan H., Ljubimov A.V., Ghiasi H. (2018). Role of Herpes Simplex Virus Type 1 (HSV-1) Glycoprotein K (gK) Pathogenic CD8(+) T Cells in Exacerbation of Eye Disease. Front. Immunol..

[61] Matundan H.H., Mott K.R., Akhtar A.A., Breunig J.J., Ghiasi H. (2015). Mutations within the pathogenic region of herpes simplex virus 1 gK signal sequences alter cell surface expression and neurovirulence. J. Virol..

[62] Mott K.R., Chentoufi A.A., Carpenter D., BenMohamed L., Wechsler S.L., Ghiasi H. (2009). The role of a glycoprotein K (gK) CD8+ T-cell epitope of herpes simplex virus on virus replication and pathogenicity. Investig. Ophthalmol. Vis. Sci..

[63] Mott K.R., Perng G.C., Osorio Y., Kousoulas K.G., Ghiasi H. (2007). A recombinant herpes simplex virus type 1 expressing two additional copies of gK is more pathogenic than wild-type virus in two different strains of mice. J. Virol..

[64] Wang S., Mott K.R., Wawrowsky K., Kousoulas K.G., Luscher B., Ghiasi H. (2017). Binding of Herpes Simplex Virus 1 UL20 to GODZ (DHHC3) Affects Its Palmitoylation and Is Essential for Infectivity and Proper Targeting and Localization of UL20 and Glycoprotein K. J. Virol..

[65] Duarte L.F., Farias M.A., Alvarez D.M., Bueno S.M., Riedel C.A., Gonzalez P.A. (2019). Herpes Simplex Virus Type 1 Infection of the Central Nervous System: Insights Into Proposed Interrelationships With Neurodegenerative Disorders. Front. Cell NeuroSci..

[66] Lathe R., Haas J.G. (2017). Distribution of cellular HSV-1 receptor expression in human brain. J. NeuroVirol..

[67] Arii J., Wang J., Morimoto T., Suenaga T., Akashi H., Arase H., Kawaguchi Y. (2010). A single-amino-acid substitution in herpes simplex virus 1 envelope glycoprotein B at a site required for binding to the paired immunoglobulin-like type 2 receptor alpha (PILRalpha) abrogates PILRalpha-dependent viral entry and reduces pathogenesis. J. Virol..

[68] Heldwein E.E., Lou H., Bender F.C., Cohen G.H., Eisenberg R.J., Harrison S.C. (2006). Crystal structure of glycoprotein B from herpes simplex virus 1. Science.

[69] Gallagher J.R., Atanasiu D., Saw W.T., Paradisgarten M.J., Whitbeck J.C., Eisenberg R.J., Cohen G.H. (2014). Functional fluorescent protein insertions in herpes simplex virus gB report on gB conformation before and after execution of membrane fusion. PLoS Pathog..

[70] Arii J., Goto H., Suenaga T., Oyama M., Kozuka-Hata H., Imai T., Minowa A., Akashi H., Arase H., Kawaoka Y. (2010). Non-muscle myosin IIA is a functional entry receptor for herpes simplex virus-1. Nature.

[71] Satoh T., Arii J., Suenaga T., Wang J., Kogure A., Uehori J., Arase N., Shiratori I., Tanaka S., Kawaguchi Y. (2008). PILRalpha is a herpes simplex virus-1 entry coreceptor that associates with glycoprotein B. Cell.

[72] Chowdhury S., Naderi M., Chouljenko V.N., Walker J.D., Kousoulas K.G. (2012). Amino acid differences in glycoproteins B (gB), C (gC), H (gH) and L (gL) are associated with enhanced herpes simplex virus type-1 (McKrae) entry via the paired immunoglobulin-like type-2 receptor alpha. Virol. J..

[73] Knebel-Morsdorf D. (2016). Nectin-1 and HVEM: Cellular receptors for HSV-1 in skin. Oncotarget.

[74] Warner M.S., Geraghty R.J., Martinez W.M., Montgomery R.I., Whitbeck J.C., Xu R., Eisenberg R.J., Cohen G.H., Spear P.G. (1998). A cell surface protein with herpesvirus entry activity (HveB) confers susceptibility to infection by mutants of herpes simplex virus type 1, herpes simplex virus type 2, and pseudorabies virus. Virology.

[75] Manoj S., Jogger C.R., Myscofski D., Yoon M., Spear P.G. (2004). Mutations in herpes simplex virus glycoprotein D that prevent cell entry via nectins and alter cell tropism. Proc. Natl. Acad. Sci. USA.

[76] Friedman G.K., Bernstock J.D., Chen D., Nan L., Moore B.P., Kelly V.M., Youngblood S.L., Langford C.P., Han X., Ring E.K. (2018). Enhanced Sensitivity of Patient-Derived Pediatric High-Grade Brain Tumor Xenografts to Oncolytic HSV-1 Virotherapy Correlates with Nectin-1 Expression. Sci. Rep..

[77] Montgomery R.I., Warner M.S., Lum B.J., Spear P.G. (1996). Herpes simplex virus-1 entry into cells mediated by a novel member of the TNF/NGF receptor family. Cell.

[78] Marsters S.A., Ayres T.M., Skubatch M., Gray C.L., Rothe M., Ashkenazi A. (1997). Herpesvirus entry mediator, a member of the tumor necrosis factor receptor (TNFR) family, interacts with members of the TNFR-associated factor family and activates the transcription factors NF-kappaB and AP-1. J. Biol. Chem..

[79] Harrop J.A., Reddy M., Dede K., Brigham-Burke M., Lyn S., Tan K.B., Silverman C., Eichman C., DiPrinzio R., Spampanato J. (1998). Antibodies to TR2 (herpesvirus entry mediator), a new member of the TNF receptor superfamily, block T cell proliferation, expression of activation markers, and production of cytokines. J. Immunol..

[80] Mauri D.N., Ebner R., Montgomery R.I., Kochel K.D., Cheung T.C., Yu G.L., Ruben S., Murphy M., Eisenberg R.J., Cohen G.H. (1998). LIGHT, a new member of the TNF superfamily, and lymphotoxin alpha are ligands for herpesvirus entry mediator. Immunity.

[81] Spear P.G., Longnecker R. (2003). Herpesvirus entry: An update. J. Virol..

[82] Shi F., Xin V.W., Liu X.Q., Wang Y.Y., Zhang Y., Cheng J.T., Cai W.Q., Xiang Y., Peng X.C., Wang X. (2020). Identification of 22 Novel Motifs of the Cell Entry Fusion Glycoprotein B of Oncolytic Herpes Simplex Viruses: Sequence Analysis and Literature Review. Front. Oncol..

[83] Wang J., Fan Q., Satoh T., Arii J., Lanier L.L., Spear P.G., Kawaguchi Y., Arase H. (2009). Binding of herpes simplex virus glycoprotein B (gB) to paired immunoglobulin-like type 2 receptor alpha depends on specific sialylated O-linked glycans on gB. J. Virol..

[84] Fournier N., Chalus L., Durand I., Garcia E., Pin J.J., Churakova T., Patel S., Zlot C., Gorman D., Zurawski S. (2000). FDF03, a novel inhibitory receptor of the immunoglobulin superfamily, is expressed by human dendritic and myeloid cells. J. Immunol..

[85] Shiratori I., Ogasawara K., Saito T., Lanier L.L., Arase H. (2004). Activation of natural killer cells and dendritic cells upon recognition of a novel CD99-like ligand by paired immunoglobulin-like type 2 receptor. J. Exp. Med..

[86] Gianni T., Salvioli S., Chesnokova L.S., Hutt-Fletcher L.M., Campadelli-Fiume G. (2013). alphavbeta6- and alphavbeta8-integrins serve as interchangeable receptors for HSV gH/gL to promote endocytosis and activation of membrane fusion. PLoS Pathog..

[87] Gianni T., Massaro R., Campadelli-Fiume G. (2015). Dissociation of HSV gL from gH by alphavbeta6- or alphavbeta8-integrin promotes gH activation and virus entry. Proc. Natl. Acad. Sci. USA.

[88] Hussein H.A., Walker L.R., Abdel-Raouf U.M., Desouky S.A., Montasser A.K., Akula S.M. (2015). Beyond RGD: Virus interactions with integrins. Arch. Virol..

[89] Agelidis A.M., Shukla D. (2015). Cell entry mechanisms of HSV: What we have learned in recent years. Future Virol..

[90] Liu X., Cohen J.I. (2015). The role of PI3K/Akt in human herpesvirus infection: From the bench to the bedside. Virology.

[91] Cheshenko N., Trepanier J.B., Stefanidou M., Buckley N., Gonzalez P., Jacobs W., Herold B.C. (2013). HSV activates Akt to trigger calcium release and promote viral entry: Novel candidate target for treatment and suppression. FASEB J..

[92] Cheshenko N., Trepanier J.B., Gonzalez P.A., Eugenin E.A., Jacobs W.R., Herold B.C. (2014). Herpes simplex virus type 2 glycoprotein H interacts with integrin alphavbeta3 to facilitate viral entry and calcium signaling in human genital tract epithelial cells. J. Virol..

[93] Lindahl U., Kusche-Gullberg M., Kjellen L. (1998). Regulated diversity of heparan sulfate. J. Biol. Chem..

[94] Shukla D., Spear P.G. (2001). Herpesviruses and heparan sulfate: An intimate relationship in aid of viral entry. J. Clin. Investig..

[95] Shukla D., Liu J., Blaiklock P., Shworak N.W., Bai X., Esko J.D., Cohen G.H., Eisenberg R.J., Rosenberg R.D., Spear P.G. (1999). A novel role for 3-O-sulfated heparan sulfate in herpes simplex virus 1 entry. Cell.

[96] Tiwari V., Clement C., Xu D., Valyi-Nagy T., Yue B.Y., Liu J., Shukla D. (2006). Role for 3-O-sulfated heparan sulfate as the receptor for herpes simplex virus type 1 entry into primary human corneal fibroblasts. J. Virol..

[97] Lycke E., Hamark B., Johansson M., Krotochwil A., Lycke J., Svennerholm B. (1988). Herpes simplex virus infection of the human sensory neuron. An electron microscopy study. Arch. Virol..

[98] Nicola A.V., Hou J., Major E.O., Straus S.E. (2005). Herpes simplex virus type 1 enters human epidermal keratinocytes, but not neurons, via a pH-dependent endocytic pathway. J. Virol..

[99] Maurer U.E., Sodeik B., Grunewald K. (2008). Native 3D intermediates of membrane fusion in herpes simplex virus 1 entry. Proc. Natl. Acad. Sci. USA.

[100] Rahn E., Petermann P., Hsu M.J., Rixon F.J., Knebel-Morsdorf D. (2011). Entry pathways of herpes simplex virus type 1 into human keratinocytes are dynamin- and cholesterol-dependent. PLoS ONE.

[101] Aggarwal A., Miranda-Saksena M., Boadle R.A., Kelly B.J., Diefenbach R.J., Alam W., Cunningham A.L. (2012). Ultrastructural visualization of individual tegument protein dissociation during entry of herpes simplex virus 1 into human and rat dorsal root ganglion neurons. J. Virol..

[102] Eisenberg R.J., Atanasiu D., Cairns T.M., Gallagher J.R., Krummenacher C., Cohen G.H. (2012). Herpes virus fusion and entry: A story with many characters. Viruses.

[103] de Duve C. (1983). Lysosomes revisited. Eur. J. BioChem..

[104] Nicola A.V., McEvoy A.M., Straus S.E. (2003). Roles for endocytosis and low pH in herpes simplex virus entry into HeLa and Chinese hamster ovary cells. J. Virol..

[105] Milne R.S., Nicola A.V., Whitbeck J.C., Eisenberg R.J., Cohen G.H. (2005). Glycoprotein D receptor-dependent, low-pH-independent endocytic entry of herpes simplex virus type 1. J. Virol..

[106] Koyama A.H., Uchida T. (1987). The mode of entry of herpes simplex virus type 1 into Vero cells. Microbiol. Immunol..

[107] Smith G.A., Pomeranz L., Gross S.P., Enquist L.W. (2004). Local modulation of plus-end transport targets herpesvirus entry and egress in sensory axons. Proc. Natl. Acad. Sci. USA.

[108] Wittels M., Spear P.G. (1991). Penetration of cells by herpes simplex virus does not require a low pH-dependent endocytic pathway. Virus Res..

[109] Tiwari V., Oh M.J., Kovacs M., Shukla S.Y., Valyi-Nagy T., Shukla D. (2008). Role for nectin-1 in herpes simplex virus 1 entry and spread in human retinal pigment epithelial cells. FEBS J..

[110] Herold B.C., WuDunn D., Soltys N., Spear P.G. (1991). Glycoprotein C of herpes simplex virus type 1 plays a principal role in the adsorption of virus to cells and in infectivity. J. Virol..

[111] Bender F.C., Samanta M., Heldwein E.E., de Leon M.P., Bilman E., Lou H., Whitbeck J.C., Eisenberg R.J., Cohen G.H. (2007). Antigenic and mutational analyses of herpes simplex virus glycoprotein B reveal four functional regions. J. Virol..

[112] Geraghty R.J., Krummenacher C., Cohen G.H., Eisenberg R.J., Spear P.G. (1998). Entry of alphaherpesviruses mediated by poliovirus receptor-related protein 1 and poliovirus receptor. Science.

[113] Suenaga T., Satoh T., Somboonthum P., Kawaguchi Y., Mori Y., Arase H. (2010). Myelin-associated glycoprotein mediates membrane fusion and entry of neurotropic herpesviruses. Proc. Natl. Acad. Sci. USA.

[114] Chowdhury S., Chouljenko V.N., Naderi M., Kousoulas K.G. (2013). The amino terminus of herpes simplex virus 1 glycoprotein K is required for virion entry via the paired immunoglobulin-like type-2 receptor alpha. J. Virol..

[115] Spear P.G., Manoj S., Yoon M., Jogger C.R., Zago A., Myscofski D. (2006). Different receptors binding to distinct interfaces on herpes simplex virus gD can trigger events leading to cell fusion and viral entry. Virology.

[116] Cai W.H., Gu B., Person S. (1988). Role of glycoprotein B of herpes simplex virus type 1 in viral entry and cell fusion. J. Virol..

[117] Desai P.J., Schaffer P.A., Minson A.C. (1988). Excretion of non-infectious virus particles lacking glycoprotein H by a temperature-sensitive mutant of herpes simplex virus type 1: Evidence that gH is essential for virion infectivity. J. Gen. Virol..

[118] Ligas M.W., Johnson D.C. (1988). A herpes simplex virus mutant in which glycoprotein D sequences are replaced by beta-galactosidase sequences binds to but is unable to penetrate into cells. J. Virol..

[119] Campadelli-Fiume G., Menotti L., Avitabile E., Gianni T. (2012). Viral and cellular contributions to herpes simplex virus entry into the cell. Curr. Opin. Virol..

[120] Connolly S.A., Jackson J.O., Jardetzky T.S., Longnecker R. (2011). Fusing structure and function: A structural view of the herpesvirus entry machinery. Nat. Rev. Microbiol..

[121] Cohen J.I. (2010). The varicella-zoster virus genome. Curr. Top. Microbiol. Immunol..

[122] Kim I.J., Chouljenko V.N., Walker J.D., Kousoulas K.G. (2013). Herpes simplex virus 1 glycoprotein M and the membrane-associated protein UL11 are required for virus-induced cell fusion and efficient virus entry. J. Virol..

[123] Klupp B.G., Nixdorf R., Mettenleiter T.C. (2000). Pseudorabies virus glycoprotein M inhibits membrane fusion. J. Virol..

[124] Jambunathan N., Charles A.S., Subramanian R., Saied A.A., Naderi M., Rider P., Brylinski M., Chouljenko V.N., Kousoulas K.G. (2015). Deletion of a Predicted beta-Sheet Domain within the Amino Terminus of Herpes Simplex Virus Glycoprotein K Conserved among Alphaherpesviruses Prevents Virus Entry into Neuronal Axons. J. Virol..

[125] Jambunathan N., Chowdhury S., Subramanian R., Chouljenko V.N., Walker J.D., Kousoulas K.G. (2011). Site-specific proteolytic cleavage of the amino terminus of herpes simplex virus glycoprotein K on virion particles inhibits virus entry. J. Virol..

[126] Beitia Ortiz de Zarate I., Cantero-Aguilar L., Longo M., Berlioz-Torrent C., Rozenberg F. (2007). Contribution of endocytic motifs in the cytoplasmic tail of herpes simplex virus type 1 glycoprotein B to virus replication and cell-cell fusion. J. Virol..

[127] Weed D.J., Nicola A.V. (2017). Herpes simplex virus Membrane Fusion. Adv. Anat. Embryol. Cell Biol..

[128] Pertel P.E., Fridberg A., Parish M.L., Spear P.G. (2001). Cell fusion induced by herpes simplex virus glycoproteins gB, gD, and gH-gL requires a gD receptor but not necessarily heparan sulfate. Virology.

[129] Terry-Allison T., Montgomery R.I., Warner M.S., Geraghty R.J., Spear P.G. (2001). Contributions of gD receptors and glycosaminoglycan sulfation to cell fusion mediated by herpes simplex virus 1. Virus Res..

[130] Terry-Allison T., Montgomery R.I., Whitbeck J.C., Xu R., Cohen G.H., Eisenberg R.J., Spear P.G. (1998). HveA (herpesvirus entry mediator A), a coreceptor for herpes simplex virus entry, also participates in virus-induced cell fusion. J. Virol..

[131] Atanasiu D., Saw W.T., Cohen G.H., Eisenberg R.J. (2010). Cascade of events governing cell-cell fusion induced by herpes simplex virus glycoproteins gD, gH/gL, and gB. J. Virol..

[132] Davis-Poynter N., Bell S., Minson T., Browne H. (1994). Analysis of the contributions of herpes simplex virus type 1 membrane proteins to the induction of cell-cell fusion. J. Virol..

[133] Visalli R.J., Brandt C.R. (1991). The HSV-1 UL45 gene product is not required for growth in Vero cells. Virology.

[134] Haanes E.J., Nelson C.M., Soule C.L., Goodman J.L. (1994). The UL45 gene product is required for herpes simplex virus type 1 glycoprotein B-induced fusion. J. Virol..

[135] Foster T.P., Melancon J.M., Baines J.D., Kousoulas K.G. (2004). The Herpes Simplex Virus Type 1 UL20 Protein Modulates Membrane Fusion Events during Cytoplasmic Virion Morphogenesis and Virus-Induced Cell Fusion. J. Virol..

[136] Melancon J.M., Fulmer P.A., Kousoulas K.G. (2007). The herpes simplex virus UL20 protein functions in glycoprotein K (gK) intracellular transport and virus-induced cell fusion are independent of UL20 functions in cytoplasmic virion envelopment. Virol. J..

[137] Han J., Chadha P., Starkey J.L., Wills J.W. (2012). Function of glycoprotein E of herpes simplex virus requires coordinated assembly of three tegument proteins on its cytoplasmic tail. Proc. Natl. Acad. Sci. USA.

[138] Debroy C., Pederson N., Person S. (1985). Nucleotide sequence of a herpes simplex virus type 1 gene that causes cell fusion. Virology.

[139] MacLean C.A., Efstathiou S., Elliott M.L., Jamieson F.E., McGeoch D.J. (1991). Investigation of herpes simplex virus type 1 genes encoding multiply inserted membrane proteins. J. Gen. Virol..

[140] Ramaswamy R., Holland T.C. (1992). In vitro characterization of the HSV-1 UL53 gene product. Virology.

[141] Foster T.P., Alvarez X., Kousoulas K.G. (2003). Plasma membrane topology of syncytial domains of herpes simplex virus type 1 glycoprotein K (gK): The UL20 protein enables cell surface localization of gK but not gK-mediated cell-to-cell fusion. J. Virol..

[142] Melancon J.M., Foster T.P., Kousoulas K.G. (2004). Genetic analysis of the herpes simplex virus type 1 (HSV-1) UL20 protein domains involved in cytoplasmic virion envelopment and virus-induced cell fusion. J. Virol..

[143] Dietz P., Klupp B.G., Fuchs W., Kollner B., Weiland E., Mettenleiter T.C. (2000). Pseudorabies virus glycoprotein K requires the UL20 gene product for processing. J. Virol..

[144] Fuchs W., Klupp B.G., Granzow H., Mettenleiter T.C. (1997). The UL20 gene product of pseudorabies virus functions in virus egress. J. Virol..

[145] Foster T.P., Chouljenko V.N., Kousoulas K.G. (2008). Functional and physical interactions of the herpes simplex virus type 1 UL20 membrane protein with glycoprotein K. J. Virol..

[146] Foster T.P., Melancon J.M., Olivier T.L., Kousoulas K.G. (2004). Herpes simplex virus type-1 (HSV-1) glycoprotein K (gK) and the UL20 protein are interdependent for intracellular trafficking and trans-Golgi network localization. J. Virol..

[147] Foster T.P., Kousoulas K.G. (1999). Genetic analysis of the role of herpes simplex virus type 1 glycoprotein K in infectious virus production and egress. J. Virol..

[148] Hutchinson L., Roop-Beauchamp C., Johnson D.C. (1995). Herpes simplex virus glycoprotein K is known to influence fusion of infected cells, yet is not on the cell surface. J. Virol..

[149] Jayachandra S., Baghian A., Kousoulas K.G. (1997). Herpes simplex virus type 1 glycoprotein K is not essential for infectious virus production in actively replicating cells but is required for efficient envelopment and translocation of infectious virions from the cytoplasm to the extracellular space. J. Virol..

[150] Foster T.P., Rybachuk G.V., Kousoulas K.G. (2001). Glycoprotein K specified by herpes simplex virus type 1 is expressed on virions as a Golgi complex-dependent glycosylated species and functions in virion entry. J. Virol..

[151] Klupp B.G., Baumeister J., Dietz P., Granzow H., Mettenleiter T.C. (1998). Pseudorabies virus glycoprotein gK is a virion structural component involved in virus release but is not required for entry. J. Virol..

[152] David A.T., Baghian A., Foster T.P., Chouljenko V.N., Kousoulas K.G. (2008). The herpes simplex virus type 1 (HSV-1) glycoprotein K(gK) is essential for viral corneal spread and neuroinvasiveness. Curr. Eye Res..

[153] David A.T., Saied A., Charles A., Subramanian R., Chouljenko V.N., Kousoulas K.G. (2012). A herpes simplex virus 1 (McKrae) mutant lacking the glycoprotein K gene is unable to infect via neuronal axons and egress from neuronal cell bodies. mBio.

[154] Hutchinson L., Johnson D.C. (1995). Herpes simplex virus glycoprotein K promotes egress of virus particles. J. Virol..

[155] Neubauer A., Osterrieder N. (2004). Equine herpesvirus type 1 (EHV-1) glycoprotein K is required for efficient cell-to-cell spread and virus egress. Virology.

[156] Mo C., Suen J., Sommer M., Arvin A. (1999). Characterization of Varicella-Zoster virus glycoprotein K (open reading frame 5) and its role in virus growth. J. Virol..

[157] Hemmings B.A., Restuccia D.F. (2012). PI3K-PKB/Akt pathway. Cold Spring Harb Perspect. Biol..

[158] Masure S., Haefner B., Wesselink J.J., Hoefnagel E., Mortier E., Verhasselt P., Eur J. (1999). Molecular cloning, expression and characterization of the human serine/threonine kinase Akt-3. Eur. J. Biochem..

[159] Hers I., Vincent E.E., Tavare J.M. (2011). Akt signalling in health and disease. Cell Signal..

[160] Brazil D.P., Hemmings B.A. (2001). Ten years of protein kinase B signalling: A hard Akt to follow. Trends BioChem. Sci..

[161] Chan T.O., Rittenhouse S.E., Tsichlis P.N. (1999). AKT/PKB and other D3 phosphoinositide-regulated kinases: Kinase activation by phosphoinositide-dependent phosphorylation. Annu. Rev. BioChem..

[162] Downward J. (1998). Lipid-regulated kinases: Some common themes at last. Science.

[163] Hemmings B.A. (1997). Akt signaling: Linking membrane events to life and death decisions. Science.

[164] Kandel E.S.N. (1999). Hay regulation and activities of the multifunctional serine/threonine kinase Akt/PKB. Exp. Cell Res..

[165] Scheid M.P., Woodgett I.R. (2001). PKB/AKT: Functional insights from genetic models. Nat. Rev. Mol. Cell Biol..

[166] Norman K.L., Sarnow P. (2010). Herpes Simplex Virus is Akt-ing in translational control. Genes Dev..

[167] MacLeod I.J., Minson T. (2010). Binding of herpes simplex virus type-1 virions leads to the induction of intracellular signalling in the absence of virus entry. PLoS ONE.

[168] Cheshenko N., Del Rosario B., Woda C., Marcellino D., Satlin L.M., Herold B.C. (2003). Herpes simplex virus triggers activation of calcium-signaling pathways. J. Cell Biol..

[169] Azab W., Gramatica A., Herrmann A., Osterrieder N. (2015). Binding of alphaherpesvirus glycoprotein H to surface alpha4beta1-integrins activates calcium-signaling pathways and induces phosphatidylserine exposure on the plasma membrane. mBio.

[170] Musarrat F., Chouljenko V., Kousoulas K.G. (2021). Cellular and Viral Determinants of HSV-1 Entry and Intracellular Transport towards Nucleus of Infected Cells. J. Virol..

